# Routine Whole-Genome Sequencing for Outbreak Investigations of *Staphylococcus aureus* in a National Reference Center

**DOI:** 10.3389/fmicb.2018.00511

**Published:** 2018-03-20

**Authors:** Geraldine Durand, Fabien Javerliat, Michèle Bes, Jean-Baptiste Veyrieras, Ghislaine Guigon, Nathalie Mugnier, Stéphane Schicklin, Gaël Kaneko, Emmanuelle Santiago-Allexant, Coralie Bouchiat, Patrícia Martins-Simões, Frederic Laurent, Alex Van Belkum, François Vandenesch, Anne Tristan

**Affiliations:** ^1^R&D Microbiology, bioMérieux, La Balme-les-Grottes, France; ^2^National Reference Center for Staphylococci, Hospices Civils de Lyon, Lyon, France; ^3^Data Analytics Unit, bioMérieux, Marcy-I’Étoile, France

**Keywords:** *Staphylococcus aureus*, whole-genome sequencing, national reference center, outbreak investigation, bioinformatics pipeline, *de novo* assembly

## Abstract

The French National Reference Center for Staphylococci currently uses DNA arrays and *spa* typing for the initial epidemiological characterization of *Staphylococcus aureus* strains. We here describe the use of whole-genome sequencing (WGS) to investigate retrospectively four distinct and virulent *S. aureus* lineages [clonal complexes (CCs): CC1, CC5, CC8, CC30] involved in hospital and community outbreaks or sporadic infections in France. We used a WGS bioinformatics pipeline based on *de novo* assembly (reference-free approach), single nucleotide polymorphism analysis, and on the inclusion of epidemiological markers. We examined the phylogeographic diversity of the French dominant hospital-acquired CC8-MRSA (methicillin-resistant *S. aureus*) Lyon clone through WGS analysis which did not demonstrate evidence of large-scale geographic clustering. We analyzed sporadic cases along with two outbreaks of a CC1-MSSA (methicillin-susceptible *S. aureus*) clone containing the Panton–Valentine leukocidin (PVL) and results showed that two sporadic cases were closely related. We investigated an outbreak of PVL-positive CC30-MSSA in a school environment and were able to reconstruct the transmission history between eight families. We explored different outbreaks among newborns due to the CC5-MRSA Geraldine clone and we found evidence of an unsuspected link between two otherwise distinct outbreaks. Here, WGS provides the resolving power to disprove transmission events indicated by conventional methods (same sequence type, *spa* type, toxin profile, and antibiotic resistance profile) and, most importantly, WGS can reveal unsuspected transmission events. Therefore, WGS allows to better describe and understand outbreaks and (inter-)national dissemination of *S. aureus* lineages. Our findings underscore the importance of adding WGS for (inter-)national surveillance of infections caused by virulent clones of *S. aureus* but also substantiate the fact that technological optimization at the bioinformatics level is still urgently needed for routine use. However, the greatest limitation of WGS analysis is the completeness and the correctness of the reference database being used and the conversion of floods of data into actionable results. The WGS bioinformatics pipeline (EpiSeq^TM^) we used here can easily generate a uniform database and associated metadata for epidemiological applications.

## Introduction

Molecular typing techniques have been pivotal in clinical and public health microbiology for uncovering the evolutionary and phylogeographic spread of medically significant microorganisms (van Belkum, 2003), which in turn supports local and global health authorities in preventing the extensive dissemination of infectious diseases (Struelens and Brisse, 2013).

Nucleic acid-based typing approaches such as pulsed field gel electrophoresis (Schwartz and Cantor, 1984), multi-locus sequence typing (MLST; Maiden et al., 1998), or the use of DNA arrays (Monecke et al., 2008; Michael Dunne et al., 2017) have been proven useful to assess epidemiological relatedness among strains of most of the health-care associated species (Sabat et al., 2013). Nevertheless, most infections in the clinical setting are caused by strains belonging to a relatively restricted number of lineages especially for highly prevalent methicillin-resistant *Staphylococcus aureus* (MRSA; Enright et al., 2002). Such strains cannot always be differentiated in sufficient detail by means of the classical DNA-based methods (Sabat et al., 2013). More recently, improvements in both the time-to-results and the affordability of whole-genome sequencing (WGS) promised to overcome this limitation by providing access to complete microbial genomes (Harris et al., 2010; Salipante et al., 2015). Both the WGS discriminatory power and its versatility helped to highlight transmission pathways intractable by the legacy Gold Standard methods (Köser et al., 2012a; Gordon et al., 2014).

Prior studies have shown the feasibility of large-scale genomic epidemiology studies in the public health and nosocomial context, leading to the generation of valuable databases covering the global epidemiology of MRSA. The same approach is also suited for solving local transmission problems involving methicillin-susceptible *S. aureus* (MSSA). Detailed research explained the dynamics of *S. aureus* transmission between patients, health care workers, and the environment (Price et al., 2017). Intra-host diversity of isolates showed less than about 100 variable nucleotide positions. Several additional studies highlighting MRSA and MSSA spreading routes have been published (Price et al., 2013; Kong et al., 2016; Ward et al., 2016; Leistner et al., 2017; Planet et al., 2017). In addition, the study by Long et al. (2014) has also shown good correlation between a resistance profile based on genotypic analysis versus phenotypic susceptibility testing similar to the findings of Gordon et al. (2014). This could be an add-on when WGS is applied to typing of staphylococci for epidemiological purposes.

To date, WGS is the sole approach that yields insight into both the macro- and the micro-evolutionary dynamics of bacterial pathogens over different periods of time and in different geographical locations. WGS is “portable” and has sufficient discriminatory power to reconstruct both intercontinental and local transmission of MRSA lineages. This will facilitate better tracking of emergent lineages within the community (Fridkin et al., 2005). Still, making sense of WGS data is impaired by the current lack of consensus methods for interpretation of data. The determination of an epidemiologically relevant single nucleotide polymorphism (SNP) cut-off value between isolates in order to identify transmission events is still cumbersome and ill-guided (Köser et al., 2012a; van Belkum, 2016). The practicability of the entire WGS workflow, from isolates to epidemiologically meaningful results, still has to be improved in order to transform WGS from a research tool into an easy-to-use approach to track and prevent infectious outbreaks (Le and Diep, 2013).

National Reference Laboratories are routinely solicited for in-depth strain characterization and typing to address epidemiological questions which are defined by progressive evolution of microbial pathogens. Here we confirm WGS to be an adequate strain characterization and typing method for *S. aureus* within the workflow of a National Reference Laboratory, by considering 131 isolates from multiple epidemiological events in France. We historically used array hybridization and *spa* typing for these isolates and here the use of WGS is added to assess its quality in comparison with our former methodology. In contrast with previous studies, we used a reference-free approach to assemble genome sequences, and inferred the evolutionary history based on SNPs detected within the core-genomes of each panel of isolates.

## Materials and Methods

### Strains

A series of MRSA and MSSA isolates (*n* = 131) from various epidemiological settings (community or hospital) and multiple genetic backgrounds [clonal complexes (CCs): CC1, CC5, CC8, CC30], referred previously to the French National Reference Center for Staphylococci, was selected (**Supplementary Table S1**).

An *S. aureus* clone is usually identified as a collection of isolates belonging to a single microbial species which share a recent common ancestor. When assessing clonality, timelines are important and continuing evolution may result in different identity levels, depending on mutational rate and on the bacterial species involved. The isolates used in this study were all characterized by DNA microarray analysis, occasionally by *spa* typing and finally by WGS. The strains are always defined by CC affiliation, absence or presence of *mec*A, and by SCCmec type. Strains may also be defined based on the absence or presence of additional characteristic genes (toxins, resistance markers, etc.). In our study, we used the SA DNA microArray (Alere) profiles and lineage assignment to identify the clonal lineages. It should be noted that the DNA microarray assignment is based on the richness of the Alere database using public sequences from both MRSA and MSSA strains, which have previously been described as new clonal lineages. Below we will describe sub-collections in more detail.

#### CC8-MRSA-IV Lyon Clone

Fifty blood culture MRSA isolates collected during a prospective multicenter study between January and July 2011 in 29 hospitals located throughout France were selected (Grundmann et al., 2014). All strains belong to the Lyon clone (CC8-MRSA-IV, *spa* type t008 or t008-related; **Supplementary Table S1**) which remains the most prevalent hospital-acquired MRSA (HA-MRSA) clone in France (Dauwalder et al., 2008).

#### CC1-MSSA Clone

Twenty-one MSSA isolates belonging to the CC1 lineage were collected during two small outbreaks geographically and temporally separated and from two sporadic cases reported to the National Reference Center for Staphylococci (**Supplementary Table S1**). The first outbreak (CC1 outbreak 1) occurred in a home for mentally disabled adult patients in the Paris area and involved 12 patients with community-acquired skin and soft tissue infections (SSTIs), including four patients with multiple recurrences between October 2013 and December 2015. We selected six isolates from these four patients. The second outbreak (CC1 outbreak 2) occurred in a French prison in 2010–2011 and involved 14 infected patients. We selected four strains from three infected patients (Bourigault et al., 2014). In addition, 11 strains from two epidemiologically unrelated patients with recurrent infections were selected: seven isolates from a first patient (patient 1) collected between 2009 and 2012 and four isolates from a second patient (patient 2) collected between 2009 and 2011. All 21 isolates were positive for Panton–Valentine leukocidin (PVL), SEA, SEH, SEK, and SEQ enterotoxins (**Supplementary Table S1**).

#### CC30-MSSA Outbreak in a School Environment

A community outbreak due to CC30 MSSA occurred in a primary school in the Val-d’Oise region (Paris area) in 2006. Over a 5-month period, 22 cases of PVL-positive *S. aureus* skin infections were confirmed in 15 primary school students and seven relatives (Carre et al., 2008, 2011). We selected 19 isolates (six from infections and 13 from colonization) collected in eight families: A (*n* = 3), B (*n* = 2), C (*n* = 4), D (*n* = 1), E (*n* = 3), F (*n* = 1), G (*n* = 4), H (*n* = 1) (**Supplementary Table S1**).

#### CC5-MRSA-I Geraldine Clone: Outbreak Among Newborns

The so-called Geraldine clone is a sequence type 5 toxic shock syndrome toxin (TSST-1)-positive SCC*mec*-I MRSA initially isolated both from community onset and health-care associated infections (Durand et al., 2006). This clone has a peculiar antibiogram and is typically resistant to penicillin, oxacillin, kanamycin, and tobramycin and intermediately resistant to fusidic acid. Forty-one strains from three outbreaks and sporadic cases were selected. The first outbreak (CC5 outbreak 1) occurred in a neonatal unit and pediatric intensive care unit (PICU) at Bordeaux University Hospital between 2010 and 2011 where 27 newborns were infected (four deaths) or colonized (Leroyer et al., 2016). The primary outbreak case was defined by a patient where MRSA with the Geraldine clone antibiogram was isolated. We selected 31 isolates from outbreak 1 (Bordeaux): 18 isolates from patients (infected or colonized), seven isolates from colonized healthcare workers, and six isolates from environmental samples. All these isolates displayed an antibiotic susceptibility profile similar to the Geraldine clone. The two other outbreaks, outbreak 2 (Limoges; Couve-Deacon et al., 2017) and outbreak 3 (Epinal), occurred in geographically independent neonatal units between 2014 and 2015 and five isolates from infected or colonized patients were selected (Limoges outbreak, *n* = 3; Epinal outbreak, *n* = 2). In addition, we analyzed four sporadic strains and the Geraldine clone reference strain described in 2003 (**Supplementary Table S1**).

### DNA Microarray Testing and *spa* Typing

#### Bacterial Genomic DNA Isolation

Chromosomal DNA was obtained from bacterial cultures grown in Brain Heart BH medium at 37°C for 3 h. After centrifugation at 3,450 × *g* for 10 min, the bacterial pellet was re-suspended in a Tris–HCl buffer (1 mM) containing Triton X-100, lysostaphin (1 mg/ml), lysozyme (10 mg/ml), and ribonuclease A. The mixture was incubated at 37°C for 30 min and proteinase K and AL buffer (DNeasy kit, Qiagen) were added. The DNA was purified using the QIAcube instrument (Qiagen, Valencia, CA, United States) according to the manufacturer’s tissue lysis protocol. The diagnostic DNA microarrays, *S. aureus* Genotyping kit 2.0 (Alere, Jena, Germany) as well as related procedures and protocols were as previously described (Monecke et al., 2008). The microarray covers 336 different target sequences corresponding to approximately 185 distinct genes and their allelic variants. The assignment of isolates to CCs was performed by comparing hybridization profiles to previously typed MLST reference strains. Spa typing was performed as previously described (Harmsen et al., 2003) and by using the Ridom SpaServer which automatically analyzes *spa* repeats and assigns *spa* types^1^.

### Whole-Genome Sequencing

We prepared sequencing libraries from 1 ng of DNA extracted using the UltraClean^®^ Microbial DNA Isolation Kit (MO BIO, Carlsbad, CA, United States). DNA quantities in extracts were controlled using a Qubit^®^ Fluorometer (Life Technologies, Carlsbad, CA, United States). Library preparation was performed with the Nextera^®^ XT DNA sample preparation kit (Illumina, San Diego, CA, United States) and Nextera^®^ XT index kit (Illumina, San Diego, CA, United States). Library validation was performed on a 2100 Bioanalyzer (Agilent Technologies, Santa Clara, CA, United States) to control the distribution of tagmented DNA. WGS was performed with an Illumina HiSeq (Illumina, San Diego, CA, United States) to generate 150-bp paired end reads. The genomes were sequenced at a minimum coverage of 30×. The sequencing raw data for each isolate were submitted to GenBank with bioProject record number PRJNA408110.

### Data Analysis

The sequencing data were processed through the EpiSeq^TM^ V1^2^ workflow. This is a reference-free approach and the workflow begins at *de novo* assembly. EpiSeq^TM^ V1 uses the A5-MiSeq open-source pipeline (Coil et al., 2015). Read cleaning consisted in removing the sequencing adapters using Trimmomatic (Lohse et al., 2012). Then, the reads were filtered and trimmed further according to quality and length criteria (**Supplementary Table S2**) using Trimmomatic and the pre-process function of String Graph Assembler SGA (**Supplementary Table S2**) (Simpson and Durbin, 2012). Finally, SGA was used to correct errors in the reads by a *k*-mer frequency-based method. After being quality filtered and error corrected, the reads were assembled by the IDBA-UD500 with *k*-mer lengths from 35 to read length -1 (Peng et al., 2013). The reads were then mapped against the assembly using BWA-MEM (Li, 2014) in order to polish the contigs at every position where base-calls differed between the mapping and the assembly. The scaffolding implemented at the end of the original A5-MiSeq pipeline was not retained since it provided no gain in subsequent marker detection.

EpiSeq^TM^ V1 also performs whole-genome-derived housekeeping gene MLST (Wg-MLST) and whole genome-*spa* (Wg-*spa*). The MLST scheme for *S. aureus* was downloaded from the public website pubmlst.org, including both the allelic sequences for the seven housekeeping genes that are routinely used and the related MLST profiles. The seven genes were located on each genome assembly using BLASTN (Altschul et al., 1990). The corresponding allele identifier was assigned by looking for an exact match between one allelic sequence in the MLST scheme and the specific gene sequence of the isolate. When no match was found, we considered the allele as new and therefore no classical MLST profile could be assigned. The Wg-MLST profiles of the strains are provided in **Supplementary Table S1**. The classical *spa* scheme for *S. aureus* was downloaded from the Ridom Spa website spaserver.ridom.de. As for Wg-MLST, we first extracted the *spa* sequence of each isolate using BLASTN against an in-house database of representative *spa* sequences. Then, we scanned the sequence for consecutive occurrence of the known short repeats and recorded the longest one as the *spa* motif for the isolate. The Wg-*spa* profiles of the strains are provided in **Supplementary Table S1**. EpiSeq^TM^ V1 uses SNPs in 2,158 core-genes (including the seven genes of the MLST scheme) to establish the phylogeny of the strains. The core-genes were selected from a pangenome constructed from 29 completely assembled *S. aureus* genomes (**Supplementary Table S3**). Orthologs from the pangenome were identified using Proteinortho5 (Lechner et al., 2011) using a cutoff of 70% of protein sequence similarity. Then the ortholog nucleic sequences were clustered with cd-hit (80% homology; Li and Godzik, 2006). For each cluster one or several representative sequences were selected with a minimum sequence homology of 90%. This core genome set was validated using 1,557 genomes from both the public domain and internal sequenced genomes. This dataset includes 924 genomes from the study of Gordon et al. (2014); 420 genomes from the NCBI Refseq genome database; 213 strains sequenced in house. Core-genes were excluded if their detection rates were low in the validation set (less than 5%) and/or the multiple-alignment of the validation sequences showed unexpected complexity (indels, inversions that could lead to alignments errors), or if they clustered with resistance or virulence markers via cd-hit (80% homology). Therefore, the housekeeping genes conferring antimicrobial resistance were not used in the phylogeny.

The genome of each isolate was then annotated by aligning the validated core gene sequences onto the genome sequences via BLASTN (Altschul et al., 1990; Camacho et al., 2009). Any hit with a percentage of identity greater than 80% and involving at least 80% of the reference sequence was kept. Then, all selected hits were clustered if their genomic coordinates onto the genome sequences overlapped by more than 10% of the length of each hit. Finally, for each cluster, only the hit with the highest alignment score was selected. This alignment score being defined as the product between the percentage of identity and the fraction of the reference sequence aligned with the genome (a score which varies between 0 and 1, 1 standing for a perfect and full-length hit). For a given comparison, we selected only those core-genes present in all the isolates to be compared and aligned their sequences using (Katoh and Standley, 2013) gene by gene. Aligned core-gene sequences were then concatenated into a final alignment and we used FastTree v1 (Price et al., 2010) with the BIONJ algorithm (Gascuel, 1997) to infer the phylogeny. EpiSeq^TM^ V1 also extracted the number of SNP in each specific core gene alignment. It calculates the number of single nucleotide differences in the alignment for each pair of strains. Indels are not taken into account, nor are they taken into account when defining the phylogeny. As a high number of continuous SNPs can indicate a recombination event, such markers were excluded from the phylogenetic analysis.

## Results

We will here separately discuss the genomic analysis of the various groups of strains as identified in Section “Materials and Methods.” By doing so, we study the strains as they would have been analyzed in the setting of a National Reference Center. In our specific examples we retrospectively compared the novel genetic analysis with the array and *spa*-sequencing analyses as they were performed in the past for these collections. Having both classical and genomic data available allows us to compare both methodologies and come to comparative quality assessment, in the end showing whether or not genome sequencing will have added value in the evaluations performed in potentially any staphylococcal reference center worldwide.

### Comparing Whole-Genome Sequencing With the Array and *spa*-Sequencing Analyses

Based on WGS, an ST could be assigned for 122 (93%) of the 131 isolates (**Supplementary Table S1**). Wg-MLST failure for nine isolates was caused by a gene present in more than one contig. Based on WGS, a *spa* type could be correctly assigned for 125 (95%) of the 131 isolates (**Supplementary Table S1**). Five isolates did not obtain a *spa* type by WGS due to the *spa* type repeats being assembled on more than one contig. In one case, the *spa* types found by the two methods were unrelated (t121/t6810). The few divergences did not have a true significance because in outbreak investigations, these isolates did cluster together with related ST and *spa* types upon SNP analysis.

### Phylogeographic Diversity of the French Dominant HA-MRSA Lyon Clone

**FIGURE 1:**
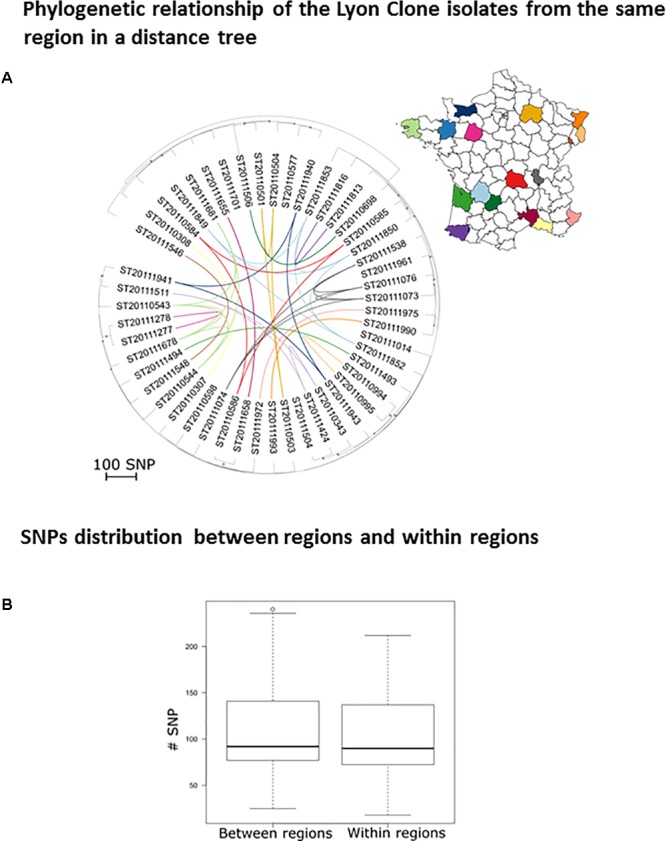
Phylogeographic diversity of the Lyon clone. **(A)** Rooted neighbor-joining tree based on the SNP distances between Lyon clone isolates (*n* = 50). The scale represents the number of SNPs in 1,524 core genes. The dots indicate nodes supported by a bootstrap score over 80, based on 100 repetitions. The lines signify clones isolated in the same region and the color code indicates the geographical origin. **(B)** Box plot of the number of SNPs between clone Lyon isolates from the same regions (intra) and between isolates belonging to different regions (inter).

The Lyon clone (ST8; SCC*mec* type IV; Dauwalder et al., 2008) has gradually replaced the gentamicin-resistant Iberian clone (ST247; SCC*mec* type I) which predominated in France in the 1990s (Lelievre et al., 1999). In order to assess if the evolutionary dynamics of this clone is now purely local or if transmissions occur between regions and hospitals, we attempted to correlate the phylogeny of 50 isolates with their geographic origin (**Figure 1A**). The absence of correlation between the region of origin and the phylogeny indicates a continuous strain flow between regions due to carriage and carriers’ mobility. Under the assumption of a lesser strain flow between regions, we should have observed a smaller genetic distance among strains from within a region than between strains from different regions. This was not the case (**Figure 1B**). For example, ST20111849 and ST20111852 isolates both originated from the same region (Périgueux), sharing the same *spa* type (t008) and toxin profile were quite far apart within the phylogenetic tree differing by 110 SNPs (**Supplementary Table S4**). In contrast, two t024 isolates (ST20110277 and ST20110278), originating from Le Mans, were genetically closely related (18 SNPs) and two t008 isolates originated from different regions, ST20110586 from Clermont Ferrand and ST20111658 from Nimes, were also genetically closely related (28 SNPs). Overall, this analysis of the French dominant HA-MRSA clone reveals a lack of large-scale geographic clustering.

### Analysis of PVL-Positive CC1-MSSA Strains Responsible for Independent Outbreaks in Mentally Disabled Patients and Prisoners as Well as Sporadic Infections

**FIGURE 2:**
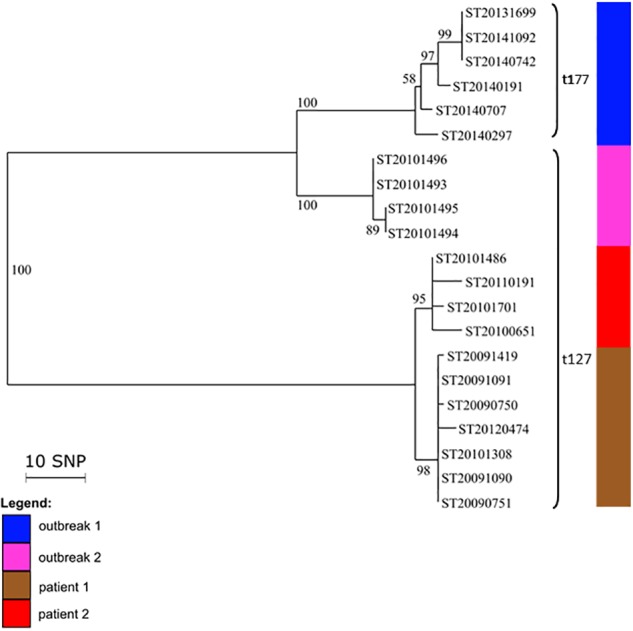
Intra- and inter-patient evolution of PVL-positive CC1 MSSA isolates responsible for outbreaks and sporadic infections. Neighbor-joining tree of 19 PVL-positive CC1 MSSA isolates that were implicated in outbreaks in a home for mentally disabled patients (outbreak 1) and in a prison (outbreak 2), and in recurrent infections in two patients (patient 1 and patient 2). The node numbers are bootstrap scores based on 100 repetitions. The scale represents the number of SNPs in 1,328 core genes.

We analyzed 21 PVL-positive CC1-MSSA isolates. All the isolates from the outbreak 1 (home for the mentally disabled) were *spa* t177 whereas the isolates from the outbreak 2 (prison) and the isolates from the sporadic cases (patient 1 and patient 2) were *spa* t127. These two *spa* types differ by only three repeats. The genetic relatedness of the 21 isolates is shown in a phylogenetic tree (**Figure 2**). Phylogenetic analysis revealed three distinct groups. The first group contained the outbreak 1 isolates that differed from each other by less than 12 SNPs. The second group contained the outbreak 2 isolates, showing even less genetic variation (<2 SNPs; **Supplementary Table S5**). Therefore, based on the topology of the tree and the length of the branches, we can confirm that the two outbreaks were not related since they were differing by 36–43 SNPs. The third group contained the isolates from the two sporadic cases (patient 1 and patient 2). The isolates from patient 1 differed by 2–10 SNPs whereas the isolates from the patient 2 differed by 0–4 SNPs. All isolates from both patients differed from each other by less than 15 SNPs, suggesting that isolates from patient 1 and patient 2 belonged to the same transmission cluster.

### Community PVL-Positive CC30-MSSA Outbreak of SSTI in a School Environment

**FIGURE 3:**
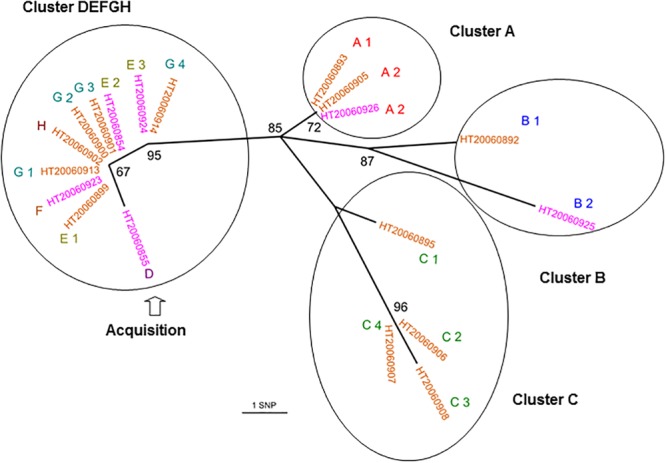
Whole-genome sequencing reveals both inter- and intra-family transmissions for a CC30-MSSA community outbreak. Unrooted joining tree of 19 PVL-positive CC30 MSSA isolates of t1848 *spa* type from eight families. Family origins are labeled with an upper case letters (A, B, C, D, E, F, G, and H) near the strain identifier and subscripts (from 1 to 4) indicate a different family member. Carriage isolates are labeled in orange and infection isolates are labeled in purple. Circles enclose clusters of isolates (0–6 SNPs). The node numbers are bootstrap scores based on 100 repetitions. The scale represents the number of SNPs in 1,328 core genes. The D isolate had an extended branch length that could explain that he was the transmitter.

We investigated 19 isolates from a community outbreak due to a PVL-positive CC30 MSSA clone, which occurred in 2006 in eight families (A–H). All the isolates had a *spa* type t1848 and were genetically closely related (0–12 SNPs; **Figure 3**). The isolates from the D, E, F, G, and H families were grouped in the phylogenetic tree (0–1 SNP) whereas the isolates from the other families were separated. The isolates from family A were indistinguishable and were separated by four to five SNPs from the DEFGH group; B1 and B2 isolates were distant by six SNPs; the isolates from family C were divergent by four to five SNPs. HT20060908 (C3) and HT20060925 (B2) isolates were the more distant isolates with 12 SNPs (**Supplementary Table S6**). The topology of the tree suggested that the first collected isolate, HT2006855 from family D, may have been directly transmitted between and within families E, F, G, and H before reaching families A, B, and C from G4 and/or E3. The fact that isolates from families A, B, and C exhibited a higher diversity (than the other families) may suggest a higher transmission and/or colonization rate within these families.

### CC5 Geraldine Clone: Outbreak Among Newborns

**FIGURE 4:**
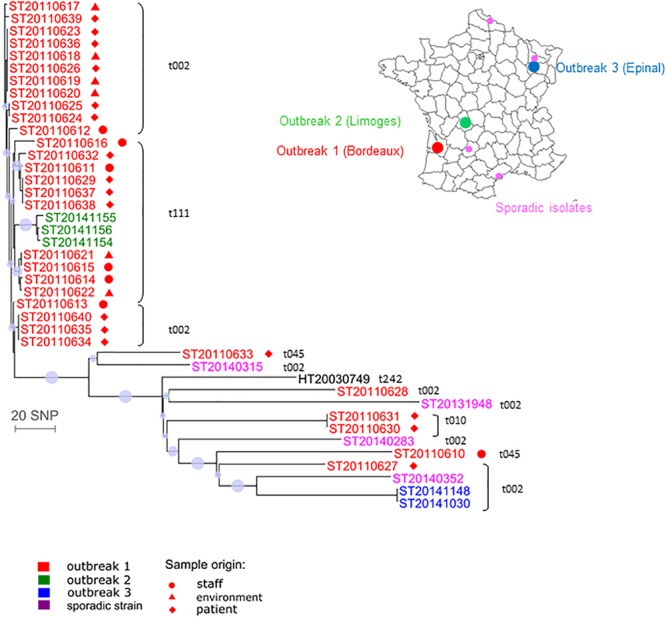
Whole-genome sequencing for investigation of outbreaks in newborns related to the Geraldine clone MRSA. Neighbor-joining distance tree computed on the SNP between 41 Geraldine clone isolates. The leaf colors refer to the outbreak or isolate origin: red stands for Bordeaux (outbreak 1), green for Limoges (outbreak 2), blue for Epinal (outbreak 3), purple for sporadic cases, black for the Geraldine clone reference strain. *Spa* type information is provided near the strain identifier. The sample origin is labeled by a symbol (see legend). The dots indicate nodes supported by a bootstrap score over 80, based on 100 repetitions. The larger the dot, the higher the score. The scale represents the number of SNPs in 2,131 core genes.

We investigated 41 CC5 isolates from three outbreaks and sporadic cases. The phylogenetic analysis revealed two distinct groups (**Figure 4**). The first group contained 25 isolates from outbreak 1 (Bordeaux, *spa* type t002 or *spa* t111) that differed from each other by less than 13 SNPs. These 25 Bordeaux isolates originated from patients, health-care workers or from the environment. The isolates from outbreak 2 (Limoges) were also included in this group and they differed by less than 22 SNPs from the Bordeaux isolates (**Supplementary Table S7**). These results suggest that this could be the same or a very closely related transmission cluster despite the fact that the *spa* type was different (t002 and t111). The second group contained six isolates from outbreak 1 (two *spa* type t002 isolates, two *spa* type t010 isolates, and two *spa* type t045 isolates), the two isolates from outbreak 3 (Epinal; *spa* t002), and four sporadic isolates and the Geraldine clone reference strain. All isolates from the second group differed from each other by a larger number of SNPs. They also differed from group 1 by 80–232 SNPs. The two Bordeaux isolates harboring a sporadic *spa* type t045 were divergent (183 SNPs) whereas two other Bordeaux isolates harboring another sporadic *spa* type (*spa* t010) were indistinguishable (these isolates were collected from a baby and his mother’s milk). We also observed an independent cluster containing the two *spa* type t002 isolates from outbreak 3. These results suggest that the group 2 isolates do not belong to the same transmission cluster.

## Discussion

In the present article, we describe the use of WGS to investigate four distinct virulent *S. aureus* lineages involved in outbreaks and/or sporadic infections in France. In each case, WGS combined with the EpiSeq^TM^ V1 bioinformatics pipeline made it possible to reveal more detailed relatedness patterns than had been observed using classical typing technologies. However, rather than fixing everything on an arbitrary genome as read mapping approaches often do, this *de novo* assembly approach offers the possibility to perform isolate comparisons either based on the predefined typing markers or via custom whole-genome comparisons.

WGS can confirm transmission events detected by conventional methods, i.e., isolates with the same *spa* type, toxin profile, and antibiogram. Here, we confirmed intra- and inter-family transmission events within a school environment by a PVL-positive CC30-MSSA clone. The better resolution of WGS enabled us to identify the first transmitter patient. We confirmed two independent outbreaks due to a PVL-positive CC1-MSSA clones, *spa* t177 and *spa* t127 that occurred in a home for the mentally disabled and in a prison. We also confirmed outbreaks with the CC5 Geraldine clone among newborns that occurred in Bordeaux, Limoges, and Epinal which were previously confirmed by conventional methods, particularly by the determination of the unusual antibiogram shared by all isolates belonging to the Geraldine clone. These results show that WGS is as good as and likely even better than the classical array hybridization and *spa* sequencing methods. In addition, WGS can also disprove transmission events falsely detected by conventional methods. Based on its single nucleotide resolution, WGS can discriminate between closely related strains whereas *spa* typing can falsely group together isolates that are genetically distant. For example, CC8 Lyon clone isolates collected in the same region (Perigueux) share the same *spa* type and toxin profile but are genetically distant (110 SNPs). This suggests that the Lyon clone population was likely introduced multiple times in each region rather than having expanded from one local ancestor resulting in a continuous dispersion of the strains between different French hospitals. This may have happened through their ability to persist within the community. Spa typing can also falsely identify patient-to-patient transmissions (Price et al., 2014). Regarding the Geraldine clone outbreak among newborns that occurred in Bordeaux, two *spa* type t002 (ST20110627 and ST20110628) have been erroneously included in the outbreak (differing by at least 152 SNPs from the Bordeaux outbreak cluster). The occurrence of the identical *spa* type t002 in the Bordeaux hospital was obviously not due to clonal spread. Therefore *spa* typing should be used carefully (Boye and Westh, 2011). This outlines that low-resolution strain typing methods are not always informative for outbreak investigations and may lead to erroneous conclusions. The 31 Bordeaux isolates were falsely considered to be part of the same outbreak since only 25 were genetically linked. This is critical for implementing measures to control an outbreak but also for avoiding inappropriate, costly, and infective ones. WGS can reveal transmission events not detected by conventional methods and epidemiological data. The power of genome sequencing to identify missing epidemiological links in nosocomial outbreaks of *S. aureus* and community outbreaks of *Mycobacterium tuberculosis* has already been demonstrated before (Gilchrist et al., 2015).

During the investigation of PVL-positive CC1-MSSA outbreaks we observed that isolates from patient 1 and patient 2 were genetically very close. These isolates shared the same *spa* type t127 but there were no known epidemiological links between the two patients except that they visited the same hospital the same year. Regarding the investigation of CC5 Geraldine clone outbreaks, we found that genetically closely related isolates from Bordeaux outbreak have two different but related *spa* types (t002 and t111). Moreover, Bordeaux outbreak isolates (*spa* t002 and *spa* t111) and isolates from the Limoges outbreak (*spa* t111) are highly related (<22 SNPs). A single base-pair change within the *spa* gene can produce two different but still highly related *spa* types. Variation of the *spa* type over time in the same strain has been already described (Boye and Westh, 2011). *Spa* t111 (26-23-17-16) is a shorter form of *spa* t002 (26-23-17-34-17-20-17-12-17-1) due to a deletion of four repeats. Thus we suspected that the Limoges cases might be linked to the Bordeaux outbreak through an undetected transmission network. However, linkage between the Bordeaux outbreak and the Limoges outbreak is based on sequence only. We do not know when and how transmission occurred and we are unaware of the epidemiological connection.

Analysis of SNPs provides a means for determining relatedness between epidemiologically linked isolates and for tracking bacterial evolution. *S. aureus* evolves primarily through point mutation, accumulating SNPs over time. Estimated mutation rates vary between 2.0 and 3.4 × 10^-6^ mutations per site per year; this equates to 5–10 SNPs in a single genome per year (Price et al., 2014). Consequently, WGS reveals the genetic relatedness of isolates at greater resolution than any more conventional technique and will identify additional temporal relationships between isolates (Price et al., 2014). Although the mutation rate is relatively well-preserved across multiple *S. aureus* lineages, knowledge of the molecular clock rates is critical in order to correctly interpret SNP differences in terms of temporal divergence of bacterial lineages (Uhlemann et al., 2014). Uhlemann et al. (2014) determined 23 SNPs as the maximum pairwise distance between pairs of isolates with clearly established epidemiological links, whereas to avoid underestimating the frequency of patient-to-patient transmission, Price et al. (2014) used a limit of >40 SNP differences to exclude recent transmission, given that SNP differences of up to 40 may be detected among *S. aureus* isolates from within a single individual (Golubchik et al., 2013). Tong et al. (2015) defined clades using an iterative procedure that assigned each sequence to a particular clade when the pairwise SNP differences (Hamming distance) were <60. During the investigation of an MRSA outbreak in a neonatal intensive care unit, Köser et al. (2012b) reported one outbreak isolate presenting a hyper-mutator phenotype with a higher number of SNPs than the other outbreak isolates. This highlights the difficulty of imposing a simple cut-off SNP value between isolates to decide whether or not they belong to the same transmission chain. Consequently this information must also be determined from the topologic characteristics of the phylogenetic tree (Köser et al., 2012b). Our data are consistent with previously published data and, interestingly, the SNP distances defining the clusters were different according to the genetic lineage and the temporal divergence, probably due to different mutation rates between lineages. The PVL-positive CC1 MSSA outbreak investigations revealed that the mean SNP number difference ranged from 0 to 15 SNPs within the three clades (2013–2014 outbreak 1 clade, 2010 outbreak 2 clade, and the clade including patient 1 and patient 2 isolates from 2009 to 2012) and from 36 to 43 SNPs between the three clades; while the CC5 Geraldine clone MRSA outbreaks among newborns revealed that the mean SNP number difference ranged from 0 to 22 SNPs within the Bordeaux–Limoges clade and from 80 to 232 between the Bordeaux–Limoges clade and the other group. Regarding the Bordeaux–Limoges clade, the maximum of 22 SNP differences between Bordeaux and Limoges isolates is fully consistent with the temporal divergence (Bordeaux outbreak 2010–2011, Limoges outbreak 2014) and the mutation rate in the core genome of three to six SNPs per year (Uhlemann et al., 2014). Moreover, the SNP distance-based clusters for all the investigated outbreaks in this study demonstrated excellent concordance with the clusters visually observed directly from the phylogenetic trees.

We observed non-determined Wg-MLST sequence types and new Wg-*spa* types due to the genome fragmentation impacting marker calling (Bartels et al., 2014, 2015). The marker calling script is unable to find markers that were split on different contigs. The Wg-MLST is particularly sensitive to this fragmentation as it involves seven different genes. However, the impact on the phylogeny is negligible as it concerns relatively few markers compared to the overall number of 2,213 markers. Then the phylogeny can be used to deduce the sequence type of the strain if the neighbors in the tree are known. Nowadays we could use additional mate-pair sequencing to reduce fragmentations. However, the potential gain (which is limited) is not worth the increase in cost and time induced by a second sequencing run. Soon, long-read sequencing (SMRT-sequencing, Pacific Bioscience, CA, United States) and Oxford Nanopore Technologies (Oxford, United Kingdom) will reduce fragmentation. EpiSeq^TM^ can be adapted to handle data originating from Pacific Bioscience and Oxford nanopore sequencers.

The key challenge will not reside in the production of the sequence data itself but instead in the ability to rapidly compute and interpret the relevant information coming from large data sets. Bioinformatics platforms have to be developed to allow intuitive data management and exploration. These data sets should conform to internationally curated standards for sets of genes and mutations that are recognized as key virulence or resistance determinants. Consequently, the greatest limitation of WGS analysis is often not the software or the sequencing technology but (i) the completeness and the correctness of the reference database being used and (ii) the conversion of floods of data into actionable results (Sabat et al., 2013; Gilchrist et al., 2015). Standard tools for whole-genome alignment can be used to compare assembled genomes to databases of microbial genomes, which are available from sources such as the NCBI and the Integrated Microbial Genomes (IMG) database (Markowitz et al., 2014). Ideally, these networks will be based on a shared bioinformatic infrastructure that links genome sequences and metadata (time, location, method of isolation, clinical details, and additional variables) with tools that help appraise the clinical and public-health relevance of any given entry (Grad and Lipsitch, 2014). However, keeping databases constantly up-to-date is difficult but remains a critical element in NGS analysis. The generic WGS bioinformatics’ workflow (EpiSeq^TM^) we use here can easily generate a uniform database and associated metadata, and therefore can be adapted to unforeseen evolution. In addition to traditional epidemiological applications, this WGS pipeline can also be effective for defining phenotypic characteristics such as virulence or antibiotic resistance.

Several studies have demonstrated the utility of WGS-based typing in prospective and retrospective outbreak studies and compared results to PFGE, MLST, *spa* typing, or other methods (Köser et al., 2012b; Harris et al., 2013; Azarian et al., 2015; Kong et al., 2016; Cunningham et al., 2017). Multiple strategies have been used for processing WGS data and determining relationships between isolates. Here, our method EpiSeq^TM^ leverages SNP analysis completed by evolutionary modeling for outbreak investigation. Other methods have focused on expanded MLST approaches (MLST+ or cg MLST). However, SNP analysis has shown particular strengths in cases where isolates are highly related: patient to patient intra-hospital transmission (Long et al., 2014) or intra-family transmission cases (outbreak of PVL-positive CC306MSSA in a school environment reported in this study).

All WGS methods yielded equivalent results, suggesting that factors other than methodology, such as cost and turnaround time, may drive the decision as to which method to use (Cunningham et al., 2017). The current costs of WGS still limit its daily use in the routine settings of reference centers and conventional methods continue to prevail for isolate’s characterization. However, the latest technical advances as well as constant cost decreases suggest that WGS will gradually outplace the classical methods and become the primary typing tool. Although *spa* typing and WGS are comparable in terms of execution time, our WGS pipeline delivers much more than just a *spa* type. It generates insights extracted from the whole genome which are produced within the same turnaround time. Moreover, SNP analysis improves the resolution even within a *spa* type. The costs of WGS are steadily decreasing and render traditional methods relatively more expensive. In real life, several classical methods need to be applied altogether in order to fully characterize a single isolate. WGS has the ability to deliver complete and unambiguous typing of different bacterial isolates. Thanks to its versatility, WGS is the all-in-one characterization method. However, replacing a well-established method by a new one has to be conducted gradually with caution in order to avoid the loss of precious historical information accumulated over the years.

## Conclusion

Throughout our retrospective study, we have demonstrated that compared to conventional typing methods, WGS provides true additional insights into the evolutionary and transmission dynamics of *S. aureus*. The usefulness of WGS for outbreak investigation is currently still limited by the lack of real-time deployment of this technology. In addition, the enormous volume of data generated by NGS technology constitutes a major challenge to microbiology laboratories lacking dedicated bioinformatic support. In this study, we moved a step forward toward improving the accessibility of WGS by using a bioinformatics pipeline based on *de novo* assembly and by the inclusion of epidemiological markers. In parallel, and independently to the changes in sequencing technology, a strong requirement for global curated databases of phylogenetic, resistance, and virulence markers is mandatory to ease both sharing of experience and data exchange between laboratories as well as between hospitals. Databasing of these markers and their associated results is the cornerstone to making EpiSeq^TM^ a credible alternative to current practices and to deliver high-quality standards to the scientific community.

## Author Contributions

GD, FV, AT, and J-BV contributed to the conception and the design of the work. FJ, MB, J-BV, GG, NM, GK, SS, ES-A, and CB contributed to the acquisition and the analysis of the data. GD, FJ, MB, and AT contributed to the interpretation of the data. GD, AT, and FV revised the work. GD, FJ, and AT wrote the article. AVB, FV, FL, and PM-S approved the final version to be published.

## Conflict of Interest Statement

The whole-genome sequencing service was provided by bioMérieux. The authors declare that the research was conducted in the absence of any commercial or financial relationships that could be construed as a potential conflict of interest.

## Footnotes

1 http://spa.ridom.de/index.html

2 https://biomerieux-episeq.com

## Supplementary Material

TABLE S1 Strain data.

TABLE S2 Data analysis parameters set.

TABLE S3 Genome list used for the pangenome construction (complete circular assembly).

TABLE S4 CC8 SNPs matrix.

TABLE S5 CC1 SNPs matrix.

TABLE S6 CC30 SNPs matrix.

TABLE S7 CC5 SNPs matrix.
