# Supplementary material for: Routine Whole-Genome Sequencing for Outbreak Investigations of Staphylococcus aureus in a National Reference Center
Source: Front Microbiol. 2018 Mar 20;9:511. doi: 10.3389/fmicb.2018.00511 (PMC5869177; doi:10.3389/fmicb.2018.00511)
Supplement: TABLE S2 — Data analysis parameters set. [file Table_2.DOC]

# Supplementary Table 2. Data analysis parameters set

**Trimmomatic parameters:**

ILLUMINACLIP:$adapter:2:30:10

LEADING:3

TRAILING:3

SLIDINGWINDOW:4:15

MILEN:36

**String Graph Assembler SGA parameters:**

Q_TRIM:25

Q_FILTER:20

Min_READ_Length:35

SGA_Dust:8
