# Supplementary material for: Routine Whole-Genome Sequencing for Outbreak Investigations of Staphylococcus aureus in a National Reference Center
Source: Front Microbiol. 2018 Mar 20;9:511. doi: 10.3389/fmicb.2018.00511 (PMC5869177; doi:10.3389/fmicb.2018.00511)
Supplement: TABLE S3 — Genome list used for the pangenome construction (complete circular assembly). [file Table_3.DOC]

# Supplementary Table 3. Genome list used for the pangenome construction (complete circular assembly).

| Strain ID | genebank ID |
| --- | --- |
| *Staphylococcus aureus* 08BA02176 | NC_018608 |
| *Staphylococcus aureus* RF122 | NC_007622 |
| *Staphylococcus aureus subsp aureus* JKD6008 | NC_017341 |
| *Staphylococcus aureus subsp aureus* COL | NC_002951 |
| *Staphylococcus aureus subsp aureus* N315 | NC_002745 |
| *Staphylococcus aureus subsp aureus* Mu3 | NC_009782 |
| *Staphylococcus aureus subsp aureus* USA300 FPR3757 | NC_007790 |
| *Staphylococcus aureus subsp aureus* Newman | NC_009641 |
| *Staphylococcus aureus subsp aureus* ED98 | NC_013450 |
| *Staphylococcus aureus subsp aureus* ED133 | NC_017337 |
| *Staphylococcus aureus subsp aureus* ST398 | NC_017333 |
| *Staphylococcus aureus subsp aureus* TW20 | NC_017331 |
| *Staphylococcus aureus subsp aureus* JKD6159 | NC_017338 |
| *Staphylococcus aureus subsp aureus* ECT R 2 | NC_017343 |
| *Staphylococcus aureus subsp aureus* TCH60 | NC_017342 |
| *Staphylococcus aureus subsp aureus* T0131 | NC_017347 |
| *Staphylococcus aureus subsp aureus* VC40 | NC_016912 |
| *Staphylococcus aureus subsp aureus* LGA251 | NC_017349 |
| *Staphylococcus aureus subsp aureus* 11819 97 | CP003194 |
| *Staphylococcus aureus subsp aureus* MSHR1132 | NC_016941 |
| *Staphylococcus aureus subsp aureus* 71193 | NC_017673 |
| *Staphylococcus aureus subsp aureus* TCH1516 | NC_010079 |
| *Staphylococcus aureus subsp aureus* JH9 | NC_009487 |
| *Staphylococcus aureus subsp aureus* MW2 | NC_003923 |
| *Staphylococcus aureus subsp aureus* Mu50 | NC_002758 |
| *Staphylococcus aureus subsp aureus* MRSA252 | NC_002952 |
| *Staphylococcus aureus subsp aureus* MSSA476 | NC_002953 |
| *Staphylococcus aureus subsp aureus* JH1 | NC_009632 |
| *Staphylococcus aureus subsp aureus* NCTC 8325 | NC_007795 |
